# Nanoparticles: An Experimental Study of Zinc Nanoparticles Toxicity on Marine Crustaceans. General Overview on the Health Implications in Humans

**DOI:** 10.3389/fpubh.2020.00192

**Published:** 2020-05-21

**Authors:** Luigi Vimercati, Domenica Cavone, Antonio Caputi, Luigi De Maria, Michele Tria, Ermelinda Prato, Giovanni Maria Ferri

**Affiliations:** ^1^Unit of Occupational Medicine, Interdisciplinary Department of Medicine (DIM), School of Medicine, University Hospital “Policlinico”, University of Bari “A. Moro”, Bari, Italy; ^2^Marine Environment and Pollution Prevention, Department of Prevention, ASL TA Health Company, Taranto, Italy; ^3^Institute for the Coastal Marine Environment of the Italian National Research Council (IAMC-CNR), Taranto, Italy

**Keywords:** nanoparticles, Zinc oxide nanoparticles (ZnO NPs), toxicity, marine crustaceans, health risks, workers, consumers, general population

## Abstract

The presence of products containing nanoparticles or nanofibers is rapidly growing. Nanotechnology involves a wide spectrum of industrial fields. There is a lack of information regarding the toxicity of these nanoparticles in aqueous media. The potential acute toxicity of ZnO NPs using two marine crustacean species: the copepod *Tigriopus fulvus* and the amphypod *Corophium insidiosum* was evaluated. Acute tests were conducted on adults of *T. Fulvus* nauplii and *C. insidiosum*. Both test species were exposed for 96 h to 5 increasing concentrations of ZnO NPs and ZnSO_4_H_2_O, and the endpoint was mortality. Statistical analysis revealed that the mean LC50 values of both ZnO NPs and ZnSO_4_H_2_O (ZnO NPs: *F* = 59.42; *P* < 0.0015; ZnSO_4_H_2_O: *F* = 25.57; *P* < 0.0015) were significantly lower for *Tigriopus fulvus* than for *Corophium insidiosum*. This result confirms that the toxic effect could be mainly attributed to the Zn ions, confirming that the dissolution processes play a crucial role in the toxicity of the ZnO NPs.

## Introduction

The number of products containing nanoparticles (NPs) or nanofibers has grown considerably. Nanotechnology involves a wide spectrum of industrial fields and offers the potential for enormous improvements in economic growth, health, technological production and environmental rehabilitation. The increasing use of nanomaterials (NMs) in consumer products has raised concerns about their potential risks for the workers, consumers and environment (1). Understanding the effects of NPs in exposed subjects is becoming a public and occupational health priority since according to some authors by 2020, the number of workers involved in the nanotechnology sector worldwide will be approximately six million (2–6). From a health perspective, animal investigations are important to extrapolate possible biomarkers of exposure and effects as well as to link the biomarkers in experimental animals to biomarkers in humans. Considering the extremely high doses frequently employed in experimental settings and the extremely low NPs-retrieved fractions in biological fluids, potential biomarkers should be validated under low doses and longer periods of treatment and related to long-term effects, such as those potentially experienced by the general and occupationally exposed population (7–10).

Given their diffusion, there is a lack of information in the consulted literature regarding the toxicity of NPs in aqueous media, and the need to implement this information has emerged. Crustaceans are important biological indicators that play a fundamental role as primary and sometimes even secondary consumers. As predators of bacteria, plants, algae, and other invertebrates, or as feeders on substrates, crustaceans have become preferential prey of large organisms, which in turn represent a large part of the human diet (11).

Extrapolating the animal data to humans by estimating an equivalent dose is necessary to lower the risk levels (8). This deficiency in information regarding NP toxicity can affect not only risk assessment but also the formulation of NPs regulations. While there are doubts and uncertainties, legislation will not be able to support the sustainable development of nanotechnologies, and the entire productive sector risks being blocked (3). In fact, competent authorities applying the principle of “*no data, no market*” (12) could prevent the use and sale of materials that are presumed to be dangerous for which there is no toxicological and ecological information.

## General Overview of the Health Implications of NPs for Workers, Consumers, General Population, and Environment

The term “nanomaterial” is defined in the European Commission (EC) recommendation [EC 2011-(696/2011)] (13) NMs are defined by the International Organization for Standardization (ISO) (ISO TS 27687) (14, 15). According to the ISO and European Union (EU) (16), NPs (single, free) can be defined as a subgroup of nano-objects, and their agglomerates (weakly bonded, embedded) and aggregates (strongly bonded, fixed) can be divided into nanofibers, nanorods, and nanoplates. The most commonly used NMs are titanium, silver, silicon, zinc, iron, and calcium NPs. NMs produced annually and used worldwide are reported in Table 1 (17), these may be present singly or in combination with other inorganic or organic molecules, such as lipids, proteins and polysaccharides.

**Table 1:** Nanomaterials NMs produced annually.

**NMs**	**Annual production (tons)**
Carbon black	9,600,000
Synthetic amorphous silica	1,500,000
Aluminum oxide	200,000
Barium titanate	15,000
Titanium dioxide	10,000
Cerium dioxide	10,000
Zinc oxide	8,000
Carbon nanotubes and nanofibers	100–3.000
Silver nanoparticles	20

*Modified from Schulte et al. (17)*.

Debia et al. (18) reports that “the 2014 Nanowerk Nanomaterials Database contains more than 3,000 commercially available NMs from over 200 suppliers worldwide” (19).

The nanotechnology-based global market is projected to achieve $3 trillion (3–5). Other authors report that over 1,800 NMs based products are currently available on the market (7, 17, 20). Available data have estimated that the total annual amount of engineered NM produced worldwide is ~11 million tons (21–23).

About NPs of anthropogenic origin, a non-exhaustive list of the main industrial sectors and products where these, especially metal oxide based, are used includes the following: construction, health care, energy, automobile and aerospace industry, chemical industry, electronics and communication, textile, biomedicine, pharmaceutical and cosmetics industries, agriculture, food industry, processing, and packaging, as well as for water treatment and environmental remediation (1, 6, 11, 24–28). In particular, NPs enter the food chain through nanofertilizers, nanopesticides, environmental pollutants or through processed foods where nanotechnology is used to modify taste and the length of time for which a product remains usable and fit for consumption (11, 29). Obviously this entails a possible risk both for workers, in farm and in the food industry, and for consumers.

NPs may be a new challenge for public health, so many national and international agencies [i.e., World Health Organization (WHO), Food and Drug Administration (FDA), ISO, EC etc.], are involved to define an acceptable risk level following their marketing and therefore define their risk assessment, management and governance (16, 30).

EU member states, including France, Belgium, Denmark, and Sweden, have established the obligation to register for the manufacturing, importation and distribution of NMs (31). In the EU, labeling the presence of NPs in consumer products is just required for food, biocides and cosmetics (32).

Moreover, in the EU Program Horizon 2020, Project NPs Pro Safe will be implemented for a “*safe by design*” concept that is to say that NMs can maintain their physicochemical properties simultaneously with an absent or low level of toxicity (30, 33).

The properties considered for evaluating the effects of NPs on health include both intrinsic (system-independent) as particles size and shape, specific surface area, porosity, hydrophobicity, water solubility/dispersion agglomeration/aggregation, chemical composition, redox potential, photocatalytic activity, and extrinsic (system-dependent) properties as density, dustiness, zeta potential, agglomeration rate and surface affinity, dissolution rate and solubility, and reactive oxygen species generation (23, 34–37). The interaction of NPs with environmental or biological matrices may determines the formation of the so-called “*protein corona*” (8, 38). This “contamination” of their surfaces is due to adhesion of reactive chemicals and biological compounds, so in this way may lead to NPs alterations such as dissolution or degradation, complexation, aggregation, or agglomeration (16).

NPs can be much more reactive than their corresponding bulk form due to the large active surface area per mass unit (24, 36, 39, 40). All these aforementioned NPs properties are important when we study the human health risk i.e., exposure (deposition and agglomeration), absorption and distribution (transport across biological barriers such as the gut epithelium, blood-brain barrier, or skin), accumulation, and toxic potency (dose-response relationships) (41).

To facilitate hazard and risk assessment, various criteria for grouping manufactured NMs have been proposed (42, 43). Overall, there is a broad consensus on these namely in terms of physical and chemical properties, toxicity and modes of action, biokinetics, interaction with the biological fluids and formation of protein corona, genotoxicity, and the bioaccumulation. This last one is very important because NPs elimination from tissues can be very slow (i.e., years) thus being a chronic irritant stimulus for the organism (10).

Various mechanisms are involved in NP-mediated toxicity, such as oxidative stress with reactive oxygen species (ROS) generation and genotoxicity through both direct and indirect action to genetic material (11, 44, 45).

By virtue of the mechanism just described, it should also be emphasized that certain nanotubes, such as asbestos fibers, show carcinogenic effects. The IARC (International Agency for Research on Cancer) has classified these as possibly carcinogenic to humans—Group 2B (46). This classification was made because of the interactions between NMs and the immune system that may result in sterile inflammatory responses like that induced by asbestos fibers (33).

Human exposure to NPs may alter heart rate and blood pressure (16, 47). NPs toxicity has also been reported in the digestive system, nervous system, kidney, liver, reproductive system, and skin and has been reported to alter body immune responses in exposed subjects (11).

To define the impact of NPs on both the ecosystem and human health, some authors suggest to study the human health impact of low-dose by long-term exposure by means of applying the hormesis concept: “biphasic dose–response relationship characterized by a low-dose stimulation and high-dose inhibition” to NPs exposure, of workers and the general population (48). The conditions of hypersusceptibility and inflammation in response to NPs should be ascertained to predict and eventually control variable adverse outcomes/pathological responses following NM exposure and to prevent a possible flare-up of chronic diseases, such as those induced by mixtures of NMs (49).

On the other hand environmental pollution of NPs can lead to interferences at food chain, and ingested NPs can possibly be translocated from the intestine into the lymphatic system and other organs (26, 41, 50). Inhalation of NPs by patients with asthma or chronic obstructive pulmonary disease, has been demonstrated to induced chronic inflammation, in the same way this type of exposure may increase allergic reactions in atopic patients (16). NPs can be transported in the blood and lymphatic system and bypass the hematoencephalic barrier via olfactory bulb (39, 51, 52). Through blood circulation, NPs can then reach other organs, i.e., liver, kidney, spleen, and hematopoietic and nervous systems (11, 51, 53).

Oral exposure may be accidental due to presence of NPs or contaminants in food, water and consumer products (54). This kind of exposure may include uptake of residues from cosmetics or dishwashing products or can occur as a consequence of the clearance of lung-transporting materials out of the lung with the mucus, which is ultimately ingested. So ingestion of MNs via food or its contact materials is the most relevant source of oral exposure (32). The European Food Safety Authority (55) distinguishes two different mode of exposure for NPs in food, namely if there is no exposure i.e., no persistence in marketed preparations or no NM migration from food to contact materials or, on the contrary, if there is complete NM transformation in food before ingestion or during digestion. However, to date, is not know the actual determinant of consumer exposure, i.e., the transfer factor, which is the fraction of the substance transferred from the product to the air, mouth or skin and represents the estimated dose of exposure (41).

The general human population environmental exposure to NPs may be due to their release by atmospheric factors from NM waste or from NMs in catalytic paints (32, 56). NPs environmental pollution may also derivate from sewage treatment plants, abrasion from tires, disposal and incineration of waste, and direct application of NPs in agriculture (32, 56).

Lastly workers could be exposed during production and processing of products containing engineered NPs (57).

Obviously, workers may be exposed for long periods and to high levels in respect to those of consumers of NM-containing products (42). In addition to inhalation and cutaneous routes workers gastrointestinal exposure may result from the mucociliary clearance of inhaled NMs, or due to lack of personal and industrial hygiene standards (58, 59).

There have been reports that among workers exposed to NPs, the risk of developing respiratory, cardiovascular and neurological disorders is increased (57). NPs may also aggravate existing diseases, such as asthma, chronic obstructive pulmonary disease and bronchitis as well as may increase inflammation and change lung function even at low engineered NPs exposure (11, 52).

Some examples of occupational exposures to NPs reported in the literature are in the dental laboratory with the use of plaster for fillings containing zinc phosphate cements with ZnO or MgO particles in the powder form (26). While upper-airway inflammation, with possible anosmia and hyposmia, it was reported in photocopier workers exposed to NPs, while in the past these pathologies were attributed to volatile organic compounds (VOCs) (51).

Nowadays, a new challenge to protect workers and the environment is represented by the study, by multidisciplinary cross fertilization, of the adverse effects of the substances at the nanoscale because of the wide and uncontrolled diffusion of NPs in the general environment, the same substances historically studied at the macroscale.

A lot of examples may be considered, regarding the adverse effects on the environment and on men of various substances studied both at macro and NPs level, so regarding xenobiotics exposure in the general population (60–62) reported stress responses in plants exposed to heavy metals or metal-containing NPs, while Gifford et al. (63) showed that novel nano enabled sorbents can reduce multiple contaminants of health concern, resulting in groundwater that was treated to drinking water standards.

Concerning air pollution and allergic diseases (64, 65) studied the prenatal exposure to urban freeway nanoparticulate matter (n PM), vehicular aerosols, that may alter neuronal differentiation, with a toxicological model on mouse. Regarding exposure to PAHs, in workers and the general population (66, 67) recently reported the combined use of ozone, carbon nano-onions with subsequent biological degradation as a means of removing PAHs from an urban runoff or a commercial waste stream. Topuz and Uyar (68) described a new method for PAH removal from aqueous solutions using silica NPs, while Celebioglu et al. (69) using nanofibers poly-cyclodextrin membranes. Zambrano-Zaragoza et al. (70) reported that nanosystems may be candidates for efficient edible coatings for food preservation, thus allowing the incorporation of antimicrobial and antioxidant ingredients. However, this implies risks of both inhalation and oral exposure for workers and for consumers. Regarding the exposure by inhalation of traffic air pollution to pathogenic agents and other ubiquitary toxic agents, as formaldehyde, for the respiratory system (71–74) studied the concentrations of charged NPs near busy roads where traffic consisted of heavy-duty diesel vehicles that typically had high particle and charge emission rates. The presence of charge on inhaled particles can influence their effect on human health (75). Regarding the respiratory health of waste collection and disposal workers (76, 77) showed that in waste incineration plants, depending on the particle sizes, the NPs present in the fly ash may produce environmental pollution.

The overall effects of occupational exposure to engineered NPs on humans and environment have not yet been well-identified (25, 30, 32, 43, 52, 78). For many NPs, to date, data on dose-response, exposure assessment and risk characterization, are insufficient to define risk management (1, 6, 7, 11, 49, 75). In a recent systematic review, only 56 occupational exposure limits (OELs) developed for engineered NMs have been reported (36, 75). As previous referred due to the interaction of NPs with environment or biologic fluids consumers, workers and environment are exposed to transformed NMs, that may have acquired different toxicity profiles (11, 79).

ECHA has released a guidance document (80) on specific methods for calculating the inhalation, oral and dermal exposures for workers and consumers (32).

There is a general consensus on the fact that “nano” size means that the exposure limits determined for substances at macro level are not necessarily valid with respect NPs in regard to occupational, environmental and public health (28).

Until health based OELs are developed and released by official regulatory agencies for biomonitoring and environmental monitoring, to define and establish regulations for the safe use of NPs and to further develop universal standardizing methods, any possible exposure for workers, consumers, and general public needs to be minimized with a conservative approach according to precautionary principles (6, 7, 10, 15, 17, 25, 32, 54, 59, 81). Understanding the effects of NPs at exposed working contexts is becoming a public and occupational health priority due to the widespread application of nano-enabled products and the increased likelihood for consumer and workplace exposures (8, 32). NPs are an environmental health problem for the general population and for the whole biota (8, 32). The study of human and environmental toxicology is required to understand the relationships between the environmental biophysical and chemical characteristics as the biological reactivity of NPs during their lifecycle, including their disposal/recycling (48, 49).

Risk assessment and regulatory programs of NPs for occupational safety, consumer safety, and safety of the general public and environment are necessary for correct risk management, i.e., risk prevention, risk mitigation, and risk communication (25). NPs will be the next challenges for the protection of occupational and environmental health. Answers should be given to the questions on “What they are—Characterization; Where they go—Destiny and their persistence; What do they do—(Re) activities” (34, 82).

## ZnO NPs

Engineered NMs are Part of NPs and Represent the Last Frontier of the Industry. The NMs Produced in This Way Can Be Made Up of a Single Type or Combination of Engineered NPs.

According to the National Science Foundation, in the next decade, the miniaturization industry will be worth $ 1 trillion (83). Among the nano-metal oxides, the highest global production is estimated for TiO_2_, SiO_2_ and ZnO (84).

Zinc oxide is an essential ingredient of many enzymes, sunscreen in which zinc oxide (ZnO) particles are added as ultraviolet (UV) light filters, cosmetics.

ZnO NPs, are used in food products as additives, supplements, containers and packaging; in the energy sector as fuels and catalysts; in consumer electronics, semiconductors, and air filter; in the pharmaceutical industry; in biomedical engineering and also in drinking water (6, 26). These NPs have UV emission capacity, conductivity and piezoelectricity, making ZnO NPs particularly interesting for applications of electronic sensors, solar photovoltaics and transducers (85).

ZnO NPs are also a very effective photographic catalyst material with excellent UV absorption and reflection properties (86).

Impact of zinc oxide on biological functions depends on its morphology, particle size, exposure time, concentration, pH, and biocompatibility. ZnO NPs, are effective against microorganisms, their action mechanism has been ascribed to their activation by light, and their ability to disintegrate the cell membrane and accumulate in the cytoplasm where causing cell death. ZnO NPs are used in biomedicine, due to their excellent biocompatibility and low toxicity, especially as anticancer and antibacterial, because of their potent ability to trigger excess reactive oxygen species (ROS) production, release zinc ions, and induce cell apoptosis. Moreover, zinc may keep the structural integrity of insulin and is used as antidiabetic treatment (87). ZnO NPs show excellent luminescent properties and have turned them into one of the main candidates for bioimaging (88).

Generally, exposure to ZnO has been linked to adverse health and environmental effects (89–92) reported oxidative DNA damage in workers exposed to metal oxide Nms. ROS may cause ZnO NPs-induced cytotoxicity and genotoxicity. ZnO NPs are more toxic than other metallic oxide NPs because of their ion-shedding ability. ZnO NPs are not very soluble in neutral solutions on the contrary in acid environments. ZnO NPs induced significant cytotoxicity in a size dependent manner. The ZnO NPs may cause neurotoxicity and probably reached the brain by olfactory system (7, 8).

Zinc oxide NPs have also been shown to induce apoptosis in colon carcinoma cells by oxidative stress leading to cytotoxicity by inflammatory responses, mitochondrial membrane alterations, and IL-8 release in cancerous cells (11, 93). Hematological alterations were reported in animals after oral or intragastric exposure to ZnO NPs (94). Chuang et al. (95) demonstrated that ZnO NPs impair cardiopulmonary functions.

Overall, the effects observed in healthy human volunteers suggest that systemic inflammation follows ZnO-Engineered NM exposure, which may be explained by either primary local inflammation of the respiratory tract/lung and secondary resorption of inflammatory markers or primary systemic inflammation due to resorbed zinc ions.

About worker's exposure (96) has shown the possible inhalation of ZnO NPs during the spray application and power sanding of common wood sealant. Inhaled ZnO NPs fumes produced by thermal cutting, welding, and other occupational activities are able to produce, after a latency period of 4–12 h, the characteristic “zinc fever” (throat irritation, coughing, metallic taste, flu-like symptoms) (97). Other studies in the past described the development of zinc fever after exposure to 5 mg/m^3^ and a slight increase in body temperature after exposure to 2.5 mg/m^3^ ZnO NPs (98, 99). Exposure to ZnO NPs confirmed the higher incidence of cardiovascular disease found in welders (7, 8). Increases in the number of blood leukocytes and the amount of highly sensitive C-reactive protein (hsCRP) due to the exposure to 1.5 mg/m^3^ ZnO NPs from the welding fumes of zinc coated steel were also reported in different studies (11, 100–102).

Other results indicated a no-observed-effect Level (NOEL) for ZnO NPs with concentrations of 1.1–1.5 mg/m^3^ contained in welding fumes with concentrations of 1.5–2.0 mg/m^3^ (PM10) (103). These findings highlight the occupational health effects for ZnO NPs-exposed workers.

The influences of ZnO nanostructures, such as nano-plate, nano-rod, and nano-flower, on various human cancer cells were studied by examining reactive oxygen species (ROS) and genotoxicity (104). They can also be cytotoxic and genotoxic to multiple types of human cells [i.e., neuronal or epithelial cells; (105)].

About the dimension of these NPs the toxicity is generally related to their chemistry not only to their size (106, 107). The small size, ease of transport within tissues/organs, ability to cross plasma membranes, are used in biomedical applications. At the same time the small size don't have increased adverse effects on skin respect to the larger ones (108, 109).

Experimental toxicological studies have shown that numerous phosphorylcholine-containing lipid (PC-CL) species were altered when exposed to high and moderate concentrations of fine ZnO NPs. The toxicity of these NM is commonly attributed to oxidative stress, inflammation and cell membrane damage caused by lipid peroxidation (108, 109). Studies on mice indicated that the liver, kidney, lung, and pancreas were target organs for the cumulative oral exposure of 50-nm nano-ZnO and might be target organs for subchronic and chronic toxicity of orally administered 50-nm ZnO (110). The mutants of yeast (Saccharomyces cerevisiae) seems to be more sensitive to the NPs' nanospecific toxicity. These effects were attributable to dissolved zinc ions from the ZnO (nano or bulk) particles. Oxidative damage and mechanical damage contributed to the toxic effects of the ZnO particles (104).

ZnO NPs, a wide-band-gap ntype semiconductor, can interact with lipopolysaccharide molecules present in the outer membrane of Escherichia coli (87), as well as produce reactive oxygen species (ROS) under UV illumination.

Inhaled ZnO NPs cause sustained renal periglomerular and interstitial inflammation through lymphocytic infiltration in Sprague-Dawley rats (111). Hou et al. (91) summarized the toxic effects of ZnO NPs in different exposure conditions on different species. Minetto et al. (112) in his review on saltwater ecotoxicology of ZnO NPs underlined criticisms and limits referred in the current studies of their toxicity. Khosravi-Katuli et al. (113) suggested that the size of ZnO NPs can influence their toxic potential as well as the release of these NPs in the aquatic environment in presence of other contaminants. Effects of ZnO NPs on marine crustaceans have been studied using saltwater microcrustacean (114, 115), linking time variable exposure (116), or different trophic level in multi NM system (117).

The aim of this study was to provide a general overview of the health implications of NPs for workers, consumers and the general population. In this perspective, because the data on the toxicity of ZnO NPs toward some marine crustaceans remain unexplored with the risks for humans related to the food chain, the purpose of the research was to evaluate the potential acute toxicity of these using two marine crustacean species, the copepod *Tigriopus fulvus* and the amphipod *Corophium insidiosum*, and to assess their mortality upon exposure to different concentrations of ZnO NPs and the effect of the “dissolution processes” on the toxicity of the ZnO NPs.

## Materials and Methods for the Experimental Study

### Test Condition

The study evaluated the toxicity of zinc oxide nanoparticles (ZnO NPs) through the execution of acute tests with two species of native crustaceans, characterized by high representativeness and diffusion in the Mediterranean area: the copepod *Tigriopus fulvus* and the amphypod *Corophium insidiosum*. Crustaceans are placed at a key trophic level in the food chain because they connect energy flows between primary producers (algae and phanerogams) and higher-level consumers (e.g., fish) and therefore also represent a powerful vehicle for the recycling of deposited pollutants in sediments.

For these reasons, crustaceans are among the most widespread model organisms in acute biotests for the evaluation of toxicity (118).

The species selected for this study belong to distinct taxonomic groups, occupy different ecological niches, and were chosen according to their ecological relevance (key species in the food chain):

- Ease of retrieval and ease of management (available and/or livestock)
- Sensitivity to different toxic compounds
- Reproducibility of the results
- Availability of standardized methods for carrying out the tests.

Acute tests were conducted on young *T. fulvus nauplii* and *C. insidiosum* adults.

The tests were repeated three times, and the concentration that resulted in the death of 50% of individuals after 96 h of exposure (LC50) was chosen as an endpoint The results were expressed as % mortality (±sd) with a 95% confidence limit. The reference method used for conducting the toxicity tests was the ISO protocol (2005) (119). The test was considered valid when the survival of the negative control was >90% [Organization for Economic Co-operation and Development (OECD) guideline 202, 2004] (120). The detailed conditions of the assay are shown in Table 2. The algal cultivation procedures are similar to those reported in previous experiences (121–126).

**Table 2:** Experimental conditions during acute toxicity tests.

**Type of test**	**Acute**	**Acute**
Crustacean test species	*Tigriopus fulvus*	*Corophium insidiosum*
Test organisms	Nauplii larvae (24–48 h)	Adult (10–14 days)
Duration of the test	96 h	96 h
Intensity of light	500–1,200 lux, cold light	500–1,200 lux, cold light
Dark light photoperiod	16 h:8 h	12 h:12 h
Dilution/control water	Filtered sea water (0.22 μm)	Filtered sea water (0.45 μm)
Salinity ‰	38 ± 2	36 ± 2
Temperature	20 ± 2	16 ± 2
pH	8 ± 0.3	6.5–8.5
Test room	Multi-compartment plates (12)	Beaker 250 ml
Incubation volume (ml)	3	200
Bodies/replication (*n*)	10	10
Replications (*n*)	3	3
Trials (*n*)	3	3
Concentrations (*n*)	5 + control	5 + control
Concentrations (ml/L)	0.125–0.25–0.5–1.0–2.0	0.4–0.8–1.6–3.2–6.4
Change solutions	Every 48 h	Every 48 h
Supply	Absent	Absent
Endpoint	Mortality	Mortality

### Test Conditions With *Tigriopus fulvus*

The algal cultures were prepared starting from algal strains belonging to the phytography of the IRSA Institute of Taranto and were placed in soil F2 with continuous oxygenation.

**Figure 1:**
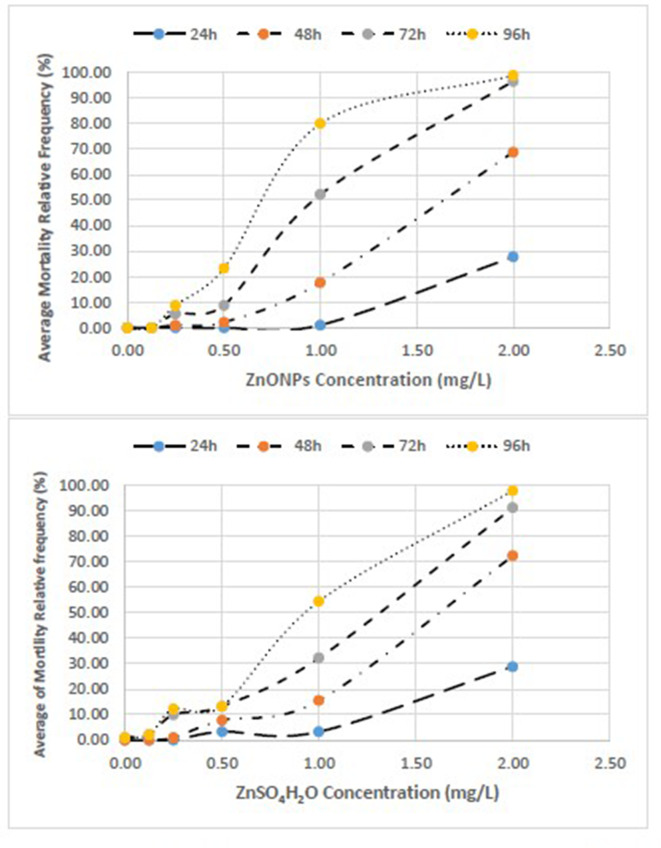
Concentration-effect relationship between Average Mortality Relative Frequency (%) of *T. fulvus* and concentration of ZnONPs and ZnSO_4_H_2_O.

The tests were carried out on synchronized stage I-II nauplii, from a culture of homogeneous organisms, hatched within 24–48 h. This choice was made because the results of previous experiments showed a great sensitivity of nauplii compared to that of adults (122, 127, 128) and a reduced variability of the results (123, 124). The algal cultures of *Tetraselmis suecica* and *Isochrysis galbana*, at a ratio of 1:2, corresponding to 1.5 × 108 and 3.0 × 108 cell/L, were added to the net/support; the plate was placed in a thermostatic environment under the same breeding conditions. Figure 1 shows the preparation of the assay (selection of females with an egg-shaped bag).

Each test was performed in a controlled environment under the same breeding conditions and repeated three times. The nauplii were exposed to the suspensions/test solutions for 96 h. The suspensions/solutions of ZnO NPs and ZnSO_4_H_2_O were prepared at scaling concentrations of 0.125–0.25–0.5–1.0–2.0 (mg/L). For each treatment and for the control, three replicates were made, each containing 10 nauplii in 3 mL of the test solution. At the end of the exposure period, the dead organisms were counted on the stereoscope. The criterion for defining mortality was the absence of movement of the appendages for an observation period of more than 20 s under light and mechanical stimulation.

*T. fulvus*, originally from the Tyrrhenian Sea (locality Calafuria, Livorno), has been cultivated for several months in the laboratory of ecotoxicology of CNR IRSA of Taranto, in seawater filtered with a 0.45 μm filter, inside 150 cm^2^ polystyrene culture flasks (0.5 L) with a ventilated cap fitted with a 0.22 mm membrane under the following conditions (121, 127, 128):

- Salinity at 38 PSU;
- Temperature of 20 ± 2°C in a thermostatic chamber;
- Photoperiod of 16L/8B at a brightness of 500–1,200 lux;
- Weekly feeding with Tetramarin®, *Tetraselmis suecica*, and *Isochrysis galbana*.

### Test Conditions With the Amphypod *Corophium insidiosum*

The tests were carried out with organisms ~10–14 days old with a size between 2 and 4 mm, i.e., young, and not yet sexually mature. This selection criterion allows the discarding of very small newborn individuals and the most resistant adults, including the ovigerous females who could release the babies during the test.

The juveniles used for the tests were collected and separated from the adults by sifting the upper 2 cm of sediment through a 500 μm mesh sieve.

Toxicity tests were performed by exposing the organisms to increasing concentrations of the two toxic agents over a period of 96 h in the absence of sediment. Ten individuals of *C. insidiosum* were randomly selected from the breeding tanks and placed in a 250 mL beaker containing 200 mL of the suspension/test solution; three replicas were performed for each concentration. For both toxicants, 5 dilutions were used with a factor of 2 starting from 0.4 (mg/L) up to 6.4 (mg/L) [0.4–0.8–1.6–3.2–6.4 (mg/L)]. The experimental conditions of temperature, salinity and photoperiod during the test are the same as those adopted during acclimatization. The details of the test are shown in Table 2. The animals used for the tests, *C. insidiosum*, were collected from an area located at Mar Piccolo in Taranto (Ionian Sea, southern Italy), which was used as a reference site due to the low levels of anthropogenic pollution (125).

The aquariums were maintained under the following conditions until the tests were carried out:

- Salinity at 36 PSU;
- Temperature of 16 ± 2°C in a thermostatic chamber;
- 12L/12B photoperiod at a brightness of 500–1,200 lux;
- Weekly feeding with 2 drops of aquarium feed (Liquefy Marine, Interpret Ltd., Dorking, UK) per liter and with the benthic microalga *Phaeodactylum tricornutum*. The animals were acclimatized to the experimental conditions for at least 7–10 days after removal from the field before the tests were performed. The sea water was changed weekly.

### Preparation of the Test Solutions

The ZnO NPs were purchased from US Research Nanomaterials, Inc., as an aqueous dispersion (20% by weight, 99.5% purity) with a nominal particle size in the range of 30–40 nm. The stock suspension of ZnO NPs (1,000 mg/L) was prepared in Milli-Q water filtered with a 0.22 μm filter from a 20% dispersion and ultrasonicated for 15' in a sonicator bath (305 W, 50–60 Hz; Soltec Ultrasonic Baths); the ZnO NPs were then stored in the dark at 4°C until the tests were performed. An intermediate suspension of 100 mg/L was subsequently prepared from a 1,000 mg/L stock suspension. The final test suspensions were prepared from the stock suspensions and sonicated immediately before each preparation in natural sea water (NSW) filtered with GFC Whatman filters (0.22–0.45 μm); the suspensions were vortexed for 5 s before the preparation of the toxicity tests without sonication. To evaluate the accuracy and reproducibility of the data under standard conditions, as well as the sensitivity of the populations employed, the ecotoxicity of the Zn ion was evaluated as a positive and solubility control. Zinc (Zn, purity ≥ 99.9%) was purchased from Sigma-Aldrich. The zinc solution was prepared by dissolving zinc sulfate (II) monohydrate (ZnSO_4_H_2_O) in Milli-Q water to obtain a metal concentration of 1,000 mg/L. Another intermediate solution of 100 mg Zn/L was subsequently prepared from the stock solution of 1,000 mg/L. Finally, the intermediate solution was further diluted in sea water filtered in calibrated flasks until the final test concentrations of the range to be tested were obtained. The identity of the compounds under examination, the CAS number, the supplier and purity are reported in Table 3.

**Table 3:** Used compound.

**Compound**	**CAS**	**Supplier**	**Purity**
ZNONPS	1314-13-2	US Research nanomaterials	99.5
ZNSO_4_H_2_O	7446-19-7	Sigma -Aldrich	99.9

The aggregation/agglomeration, of ZnO particles across the concentrations, applied in the medium (NSW) and the effective size of ZnO NPs was evaluated by means a Dynamic Light Scattering instruments (NICOMP™ 380 DLS Particle Size Analyzer, PSS, FL, USA) and by Dispersion Analyser LUMISizer (LUM GmbH, Berlin), at time 0 and after 48 h. Moreover, for DLS, size distributions were calculated by NICOMP™ cumulants analysis, that selects the best fitting distribution and calculate the intensity-weighted diameters (± standard deviation). Samples were read twice after 5 min for each sample. For LUM the particle size distributions were calculated by using SEPView 6.3 (LUMiSizer software). Zeta (ζ-) potential of the ZnO NPs dispersed in NSW was also measured by DLS; measurements were carried out in triplicate, each consisting in 5 runs.

The dissolved Zn concentrations of ZnO NPs and ZnSO_4_ suspensions test were measured by Inductively Coupled Plasma Mass Spectrometry (ICP-MS) (126).

The semistatic tests were conducted in the absence of a power supply; the suspensions/test solutions were renewed every 48 h. The water quality parameters (temperature, pH, dissolved oxygen, and salinity) were measured at the beginning and end of each test to ensure the acceptance criteria of toxicity tests (ASTM 1993, ISO 2005) (119, 129). The results are expressed as a percentage of the effect with respect to that of the control. The significance of the data obtained was evaluated on the basis of the difference between the sample and the control using Student's *t*-test for paired data. The tested matrix was judged to be toxic when the difference between the sample and control was statistically significant (*p* < 0.05). The Microsoft EXCEL program was used for recording the data and graphing the outcomes.

Each experiment was repeated three times with three replicates (*n* ≥ 9). The tests were considered acceptable when the control mortality was 0%. A mortality value <10% among the negative controls, conducted on water only, was included in the acceptability parameters. At first they were evaluated absolute and relative mortality frequencies of both the *T. fulvus* and *C. insidiosum* at 24, 48, 72, and 96 h for both ZnONPs and ZnSO_4_H_2_O. Were also built concentration-effect curves.

Analysis of variance (ANOVA) was used to perform a statistical comparison between the samples to evaluate if the toxicant used (zinc) had a significant effect on the responses of the test organisms compared to those in the control group; two species were used, and two toxicants were compared. When significant differences were found (*p* < 0.05), the *post-hoc* Tukey test was used for multiple comparisons between different concentrations and control. The assumptions of normality and homogeneity of the variances were evaluated by means of the Kolmogorov-Smirnov test and Levene's test, respectively. Statistical elaborations were performed with Statgraphics Version 5 Plus software (Statistical Graphic Corporation, Manugistics^TM^, Rockville, MD, USA) and Stata 15 software was also used for analysis (StataCorp. Stata: Release 15. Statistical Software. College Station, TX, USA: StataCorp LP; 2017).

The values of LC50 with 95% confidence limits were determined using the Litchfield-Wilcoxon method (130). Finally, reproducibility, acceptability and stability of the estimates of the three tests in both the groups of *T. fulvus* and *C. insidiosum* were based on the evaluation of the dimensions of CL95% and Variation Coefficient (VC).

## Results

**Figure 2:**
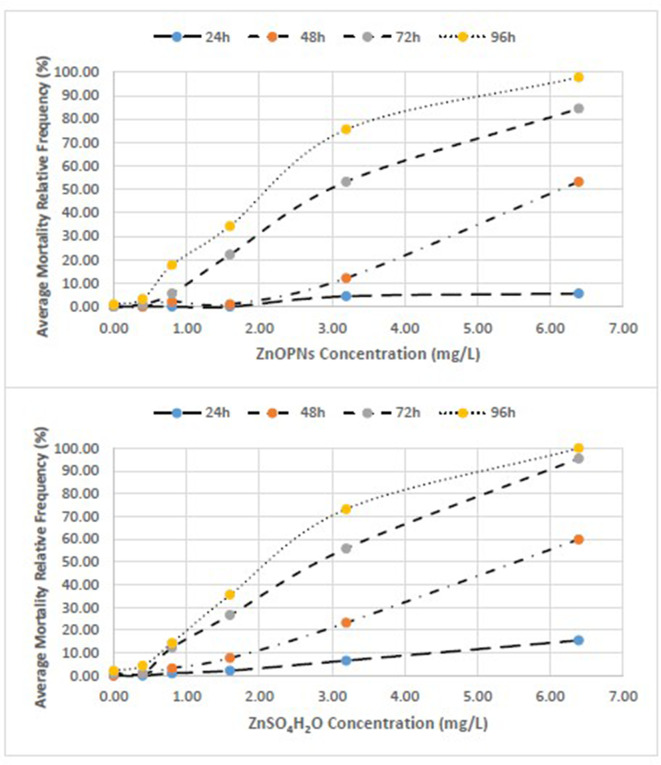
Concentration-effect relationship between Average Mortality Relative Frequency (%) of *C. insidiosum* and concentration of ZnONPs and ZnSO_4_H_2_O.

**Figure 3:**
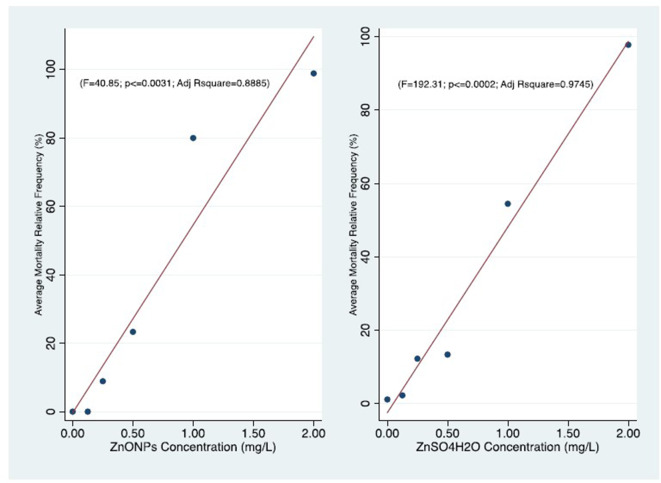
Comparison of concentration-effect curves of *T. fulvus* after 96 h of exposure to ZnONPs (*r* = 0.95; *P* ≤ 0.031) and ZnSO_4_H_2_O (*r* = 0.98; *P* ≤ 0.002).

**Figure 4:**
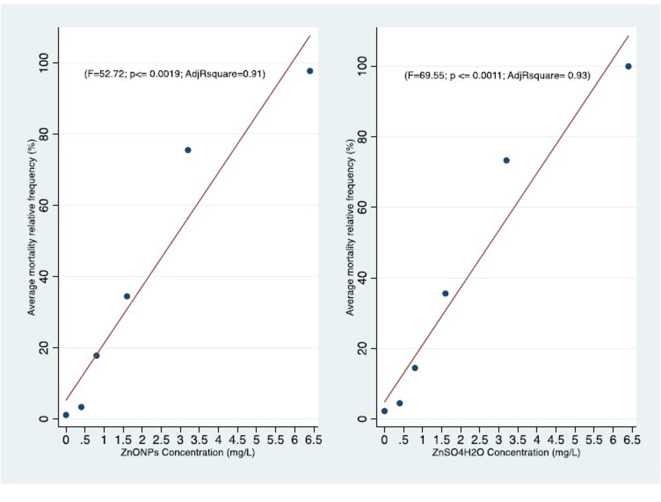
Comparison of concentration-effect curves of *C. insidiosum* after 96 h of exposure to ZnONPs (*r* = 0.96; *P* ≤ 0.019) and ZnSO_4_H_2_O (*r* = 0.97; *P* ≤ 0.0011).

We clearly reported the *absolute frequencies of deaths (n)* for the three replicates of each test and the estimates of mortality based on the three *test averages of the relative frequencies (%)* (Tables 4–6). We also represented the *concentration-effect curves*, showing their trends, by time of exposure (24–96 h) (Figures 1, 2) and by different compounds (Figures 3, 4).

**Table 4:** Mortality (relative frequencies of deaths [%]) of *T. fulvus* by time and different concentrations.

**Concentrations (mg/L)**	**ZnONPs (mgZnO/L)**					**ZnSO**_****4****_**H**_****2****_**O (mgZn/L)**				
	**1th trial (*n* = 30)**	**2nd trial (*n* = 30)**	**3rd trial (*n* = 30)**	**Means**	***SD***	**1th trial (*n* = 30)**	**2nd trial (*n* = 30)**	**3rd trial (*n* = 30)**	**Means**	***SD***
	**24 h**					**24 h**				
Controls	0.00	0.00	0.00	0.00	0.00	0.00	0.00	0.00	0.00	0.00
0.125	0.00	0.00	0.00	0.00	0.00	0.00	0.00	0.00	0.00	0.00
0.25	0.00	0.00	0.00	0.00	0.00	0.00	0.00	0.00	0.00	0.00
0.5	0.00	0.00	0.00	0.00	0.00	3.33	6.66	0.00	3.33	3.33
1	3.33	0.00	0.00	1.11	1.92	3.33	6.66	0.00	3.33	3.33
2	26.67	23.33	33.33	27.78	5.09	33.33	20.00	33.33	28.89	7.70
	**48 h**					**48 h**				
Controls	0.00	0.00	0.00	0.00	0.00	0.00	0.00	0.00	0.00	0.00
0.125	0.00	0.00	0.00	0.00	0.00	0.00	0.00	0.00	0.00	0.00
0.25	3.33	0.00	0.00	1.11	1.92	3.33	0.00	0.00	1.11	1.92
0.5	3.33	3.33	0.00	2.22	1.92	10.00	10.00	3.33	7.78	3.85
1	13.33	20.00	20.00	17.78	3.85	6.56	23.33	16.66	15.55	8.39
2	63.33	56.67	86.67	68.89	15.75	86.66	66.66	63.33	72.22	12.62
	**72 h**					**72 h**				
Controls	0.00	0.00	0.00	0.00	0.00	3.33	0.00	0.00	1.11	1.92
0.125	0.00	0.00	0.00	0.00	0.00	6.66	0.00	0.00	2.22	3.85
0.25	6.67	10.00	0.00	5.56	5.09	16.66	10.00	3.33	10.00	6.67
0.5	10.00	16.67	0.00	8.89	8.39	16.66	16.66	6.66	13.33	5.77
1	40.00	50.00	66.67	52.22	13.47	40.00	36.66	20.00	32.22	10.71
2	100.00	90.00	100.00	96.67	5.77	100.00	90.00	83.33	91.11	8.39
	**96 h**					**96 h**				
Controls	0.00	0.00	0.00	0.00	0.00	3.33	0.00	0.00	1.11	1.92
0.125	0.00	0.00	0.00	0.00	0.00	6.66	0.00	0.00	2.22	3.85
0.25	6.67	10.00	10.00	8.89	1.92	16.66	16.66	3.33	12.22	7.70
0.5	30.00	16.67	23.33	23.33	6.67	16.66	16.66	6.66	13.33	5.77
1	86.67	73.33	80.00	80.00	6.67	53.33	66.66	43.33	54.44	11.70
2	100.00	96.67	100.00	98.89	1.92	100.00	100.00	93.33	97.78	3.85
	Tukey test (96 h): *F* = 272; *p* ≤ 0.000					Tukey test (96 h): *F* = 101.19; *p* ≤ 0.000				

**Table 5:** Mortality (relative frequencies of deaths [%]) of *C. insidiosum* by time and different concentrations.

**Concentrations (mg/L)**	**ZnONPs (mgZnO/L)**					**ZnSO**_****4****_**H**_****2****_**O (mgZn/L)**				
	**1th trial (*n* = 30)**	**2nd trial (*n* = 30)**	**3rd trial (*n* = 30)**	**Means**	***SD***	**1th trial (*n* = 30)**	**2nd trial (*n* = 30)**	**3rd trial (*n* = 30)**	**Means**	***SD***
	**24 h**					**24 h**				
Controls	0.00	0.00	0.00	0.00	0.00	0.00	0.00	0.00	0.00	0.00
0.4	0.00	0.00	0.00	0.00	0.00	0.00	0.00	0.00	0.00	0.00
0.8	0.00	0.00	0.00	0.00	0.00	0.00	3.33	0.00	1.11	1.92
1.6	0.00	0.00	0.00	0.00	0.00	0.00	6.66	0.00	2.22	3.84
3.2	6.67	0.00	6.67	4.44	3.85	3.33	6.66	10.00	6.66	3.33
6.4	3.33	3.33	10.00	5.56	3.85	10.00	23.33	13.33	15.55	6.93
	**48 h**					**48 h**				
Controls	0.00	0.00	0.00	0.00	0.00	0.00	0.00	0.00	0.00	0.00
0.4	0.00	0.00	0.00	0.00	0.00	0.00	3.33	0.00	1.11	1.92
0.8	0.00	3.33	3.33	2.22	1.92	3.33	6.67	0.00	3.33	3.33
1.6	0.00	0.00	3.33	1.11	1.92	3.33	16.67	3.33	7.78	7.70
3.2	6.67	13.33	16.67	12.22	5.09	10.00	36.67	23.33	23.33	13.33
6.4	40.00	56.67	63.33	53.33	12.02	53.33	63.33	63.33	60.00	5.77
	**72 h**					**72 h**				
Controls	0.00	0.00	0.00	0.00	0.00	0.00	3.33	0.00	1.11	1.92
0.4	0.00	3.33	0.00	1.11	1.92	0.00	3.33	0.00	1.11	1.92
0.8	6.67	3.33	6.67	5.56	1.92	6.67	23.33	6.67	12.32	9.62
1.6	10.00	20.00	36.67	22.22	13.47	23.33	30.00	26.67	26.67	3.33
3.2	30.00	53.33	76.67	53.33	23.33	60.00	66.67	53.33	56.00	6.67
6.4	66.67	90.00	96.67	84.44	15.75	96.67	96.67	93.33	95.56	1.92
	**96 h**					**96 h**				
Controls	0.00	0.00	3.33	1.11	1.92	0.00	6.67	0.00	2.22	3.85
0.4	0.00	3.33	6.67	3.33	3.33	0.00	6.67	6.67	4.44	3.85
0.8	16.67	16.67	20.00	17.78	1.92	10.00	23.33	10.00	14.44	7.70
1.6	30.00	26.67	46.67	34.44	10.72	36.67	40.00	30.00	35.56	5.09
3.2	66.67	73.33	86.67	75.56	10.18	83.33	70.00	66.67	73.33	8.82
6.4	93.33	100.00	100.00	97.78	3.85	100.00	100.00	100.00	100.00	0.00
	Tukey test (96 h): *F* = 113; *p* ≤ 0.000					Tukey test (96 h): *F* = 150; *p* ≤ 0.000				

**Table 6:** Comparison of Lethal Concentration 50 (LC50) (mg/L) at 96 h of the two species of the studied crustaceans by different compounds.

		***Tigriopus fulvus***	***Corophium insidiosum***	F^*^	p^*^
ZnONPs LC50 (CL 95%)	1th trial	0.55 (0.42–0.73)	2.18 (1.76–2.69)		
	2nd trial	0.67 (0.54–0.85)	1.65 (1.35–2.00)		
	3rd trial	0.57 (0.47–0.69)	1.42 (1.18–1.72)		
	Mean	0.60 (0.47–0.76)	1.75 (1.43–2.13)	59.42	0.0015
	*SD*	0.06	0.38		
	*VC*	0.11	0.22		
ZnSO_4_H_2_O LC50 (CL 95%)	1th trial	0.63 (0.49–0.79)	1.72 (1.41–2.09)		
	2nd trial	0.60 (0.50–0.71)	1.47 (1.18–1.82)		
	3rd trial	0.88 (0.65–1.18)	1.71 (1.35–2.16)		
	Mean	0.70 (0.55–0.89)	1.63 (1.35–1.75)	25.57	0.0072
	*SD*	0.15	0.14		
	*VC*	0.22	0.08		

* *These values were estimated by means of the Litchfield-Wilcoxon method. Were reported means, standard deviations (SD), variation coefficient (VC), and 95% confidence intervals (LCI-UCI)*.

The observed similar LC50 levels of soluble salts and the particles, may be due to the ZnO NPs partial dissolution so the concentrations of free Zn^2+^ in the ZnO NPs solutions need to be measured to understand the effects of ions dissolution at different concentrations of the particles, which is important in deciding the toxic impact (131). Zinc is an essential trace element for biological organisms, but it is also known that can cause cellular damage at high concentrations. Regarding the toxic effects of ZnO NPs, some studies attributed the toxicity to the Zn ions (Zn^2+^) released from the NPs into the solution. On the other hand, the findings of other studies imply that Zn^2+^ cannot account entirely for the toxicity of ZnO NPs. It is important to understand dissolution rate of the NP in order to quantify potential for toxicological effects following exposure. The extent of this dissolution is based on a number of factors including particle size, coating, and medium. In seawater, for example, the solubility of ZnO NPs can be more than twice that of micron-size ZnO (132). Therefore, they are unlikely to remain of nano-sized in exposures to marine organisms. They are likely to aggregate with increasing salinity, thereby reducing surface area for dissolution. In this study, the dissolved Zn concentrations of ZnO NPs and ZnSO_4_ suspensions test were measured according to Prato et al. (126). The nominal ZnO NPs e Zn SO_4_ concentrations in all treatments used did not differ significantly from that measured. It is important to understand dissolution rate of the NP in order to quantify potential for toxicological effects following exposure. The analysis of the samples highlighted that ZnO NPs showed a rapid tendency to aggregate, already in the first hours of the measurement, at the lowest test concentrations (no NPs detected at both T0 and T48h). After 48 h, aggregates slight increased their size, compared to zero time, passing from 110 ± 20 nm up to 140 ± 20 nm. The aggregates showed different sizes suggesting that solution/suspension contained nano-(micro-)sized fractions potentially able to interact with biota.

The low values of the standard deviations and the coefficient of variation of the means of all the tested species and compounds and the small value of 95% CL (Table 6) confirmed the reproducibility and therefore the acceptability of the three tests.

### Acute Toxicity Test (96 h) With *T. fulvus*

The average relative mortality (%) of the nauplii after 24 h of exposure to the ZnO NPs ranged from 0.0 ± 0.0 at the lowest concentration of 0.125 mg/L to 27.8 ± 5.09 at the highest concentration of 2.00 mg/L. After 96 h of exposure, the mortality estimates increased, reaching a value of 98.9 ± 1.92 at 2.00 mg/L. The mortality estimates observed among the nauplii exposed to Zn sulfate (ZnSO_4_H_2_O) for 24 h were 0.0 ± 0.0 at the lowest concentration of 0.125 mg/L and 28.89 ± 7.70 at the concentration of 2.00 mg/L. After 96 h of exposure, the mortality estimates increased, reaching a value of 97.78 ± 3.85 at 2.00 mg/L (Table 4).

The concentration-effect relationship between the average of mortality relative frequency (%) of *T. fulvus* by different experimental times and different exposure are reported in Figure 1.

With respect to the negative control, significant effects were observed as early as 24 h at the highest concentration of 2.00 mg/L for both toxicants. At 96 h, the mortality estimates were significant, starting from 0.25 mg/L with ZnO NPs and from 0.50 mg/L with ZnSO_4_H_2_O (Supplementary Tables 1, 2).

The mortality values obtained for Zn sulfate (ZnSO_4_H_2_O) were similar to those obtained for the ZnO NPs.

With respect to the negative controls at 96 h of exposure, the observed variance of the mortality estimates of the highly exposed groups, evaluated by means of the Tukey test, was significant for ZnO NPs (*F* = 272.00; *p* ≤ 0.000) and ZnSO_4_H_2_O (*F* = 101.19; *p* ≤ 0.000). The exposed to the first compound showed a higher significant variability between groups compared with the same variability between the groups exposed to the second compound as certified by the Fisher values [*F* = 272; *p* < 0.000 vs. *F* = 101.19; *p* < 0.000; Table 4].

We observed that the mortality curves of the two different compounds had a similar trend according to the concentrations tested (Figure 3). The mean LC50 values obtained for the two forms of zinc were similar [ZnO NPs: 0.60 ± 0.06 mg/L; ZnSO_4_H_2_O: 0.70 ± 0.15 mg/L] and were not significantly different from each other (Table 6). The low values of standard deviation and coefficient of variation and the small confidence interval of LD50 for the ZnO NPs (*S.D*. = 0.06; *CV* = 0.11; and *C.I*. = 0.27) and ZnSO_4_H_2_O (*S.D*. = 0.15; *CV* = 0.22; and *C.I*. = 0.34) confirmed the reproducibility, acceptability and stability of the estimates of the three tests in the group of *T. fulvus*.

The Pearson r is highly significant in both the *T. fulvus* exposure (ZnONPs, ZnSO_4_H_2_) effects relationships. This indicate very strong correlations. The strength of those result is supported also by the linear regression significance of the Adjusted determination coefficient (*R*^2^) for same variables (Figure 3).

### Acute Toxicity Test (96 h) With *C. insidiosum*

The average relative mortality (%) (χ ± δ) of the *C. insidiosum* obtained with Zn sulfate (ZnSO_4_H_2_O) was slightly higher than that obtained with the ZnO NPs. In fact, the mortality estimates of the animals exposed to the ZnO NPs for 24 h varied from 0.00 ± 0.00 at the lowest concentration of 0.4 mg/L to 5.5 ± 3.85 at the highest concentration of 6.4 mg/L; after 96 h of exposure, mortality reached 97.8 ± 3.85 at 6.4 mg/L. Among the animals exposed to Zn sulfate (ZnSO_4_H_2_O), the estimates ranged from 0 ± 0 at the lowest concentration of 0.4 mg/L to 15.55 ± 6.93 at the highest concentration of 6.4 mg/L; after 96 h of exposure, the mortality estimates increased, reaching a value of 100.00 ± 0.00 at 6.4 mg/L. At 96 h, with respect to that of the negative controls, the observed variance of the mortality estimates of the highly exposed groups, evaluated by means of the Tukey test, is significant for the ZnO NPs (*F* = 113; *p* ≤ 0.000) and ZnSO_4_H_2_O (*F* = 150; *p* ≤ 0.000; Table 5). The concentration-effect curves reported an increase in the mortality estimates with increasing zinc concentrations and increasing exposure time. The observed trends are very similar for both the ZnO NPs and ZnSO_4_H_2_O and the concentration-effect relationship between Average Mortality Relative Frequency (%) of *C. insidiosum* and concentrations of ZnONPs and ZnSO_4_H_2_O was reported in Figure 2.

The mortality values obtained for ZnSO_4_H_2_O were similar to those obtained for the ZnO NPs. With respect to those observed for the negative control, significant effects were observed after 24 h of exposure starting from the 3.2 mg/L concentration with both toxic substances; and after 96 h, the differences were significant starting from 0.8 mg/L (Table 5).

Statistical analysis of the mortality rates for each concentration tested, after 96 h of exposure, did not show significant differences between the two forms of zinc. In fact, we observed that the two mortality curves have a similar trend according to the tested concentrations (Figure 4). The mean LC50 values obtained for the two forms of zinc were similar [ZnO NPs: 1.75 ± 0.39 mg/L; ZnSO_4_H_2_O: 1.63 ± 0.14 mg/L] and were not significantly different from each other (Table 6). The low values of standard deviation and coefficient of variation and the small confidence interval of LC50 for the ZnO NPs (*S.D*. = 0.38; *CV* = 0.22; and *C.I*. = 0.70) and ZnSO_4_H_2_O (*S.D*. = 0.14; *CV* = 0.08; and *C.I*. = 0.40) confirmed the reproducibility, acceptability and stability of the estimates of the three tests in the group of *C. insidiosum*.

The Pearson *r* is highly significant in both the *C. insidiosum* exposure (ZnONPs, ZnSO_4_H_2_) effects relationships. This indicate very strong correlations. The strength of those result is supported also by the linear regression significance of the Adjusted determination coefficient (*R*^2^) for same variables (Figure 4).

### Comparison Between the Two Test Species

Statistical analysis revealed that the mean LC50 for *Tigriopus fulvus* was significantly lower than that for *Corophium insidiosum* for both the ZnO NPs and ZnSO_4_H_2_O (ZnO NPs: *F* = 59.42; *p* < 0.0015; ZnSO_4_H_2_O: *F* = 25.57; *p* < 0.0015; Table 6).

## Discussion: the Study on Marine Crustaceans

Nanomaterials and nanotechnologies are a thriving industrial sector destined to grow due to the increasing amount of investments it is able to attract. The increase in the production and application of NPs means that more of them are released into the marine environment, which represents the final pollutant receptor.

Despite the increased use of NMs in recent years, the volumes produced and used in different applications are not known, and the resulting emissions in the environment and their impact, fate and consequences on human health are not known. These gaps do not allow an exact assessment of the risk.

The progress made in the last decade by research in the development and application of nanomaterials has helped to define a framework of policies able to support the safe and responsible development of nanotechnologies, which has emerged as an increasingly pressing issue.

That is, the need to support the growth of the nanotechnology sector was introduced in order to maximize the benefits both in economic terms and in improving the quality of life while limiting the potential risks to health and safety, and a link between these emerging technologies and their continuous evolution was established.

For the safe and responsible development of nanotechnologies, the protection of the health and safety of the environment and of the workers involved in such processes assumes a specific relevance due to the particular conditions (levels, modalities, and times) of exposure.

The results of this study were intended to be used as ecotoxicological information, showing the toxicity of ZnO NPs that enter the food chain, on two model organisms characterized by high representativeness and diffusion in the Mediterranean area, the copepod *Tigriopus fulvus* and the amphypod *Corophium insidiosum*.

Experimentation with the two selected test species has shown that to test the toxic effects of ZnO NPs, exposure times longer than 24 h are more suitable than shorter exposure times. In fact, both tested zinc forms exhibited moderate 24 h toxicity, while the effects increased considerably with increasing exposure. The two species showed different sensitivities: *Corophium insidiosum* was less sensitive than *Tigriopus fulvus* toward both forms of zinc.

These results confirm that since the sensitivity of animal and plant communities to pollutants varies significantly from one species to another, a single experimental organism or model is not able to represent the complex variety of responses to stressors (133, 134) and that the use of a single test produces results with a high level of uncertainty.

Moreover, the results obtained have shown the existence of positive relationship between the effects of toxicity found and the concentration of NPs and between the effects of toxicity and observation times. Both species showed, when exposed to the two forms of zinc (ZnO NPs and ZnSO_4_), an absence of significant differences between the LC50 values, showing the same trend with respect to time and dose. This result confirms that the toxic effect could be mainly attributed to the Zn ions, confirming that the dissolution processes would a crucial role in the toxicity of the ZnO NPs (113, 126, 135, 136). Once introduced into the aquatic environment, the NPs undergo a series of processes (aggregation, sedimentation, biological degradation, and dissolution) that modify their destiny and lead to the formation of both potentially toxic aggregates and metal ions. These processes are influenced not only by multiple environmental factors, such as pH, salinity, and the presence of organic substances, but also by the structural characteristics of the particle, which include shape, size, morphological substructure of the substance (crystallinity, porosity, and surface roughness), chemical properties of the coarse material, solubility, dispersion state, area, and surface charge (115, 137–139).

Moreover, the differences in experimental procedures, particularly regarding the preparation of the NP suspension (presence/absence of solubilization vehicles, filtering, centrifugation, sonication), can produce results that are sometimes not very comparable, making the comparison complicated (132).

The results of the acute tests on *T. fulvus* reported in this study are comparable to those found in the literature. Wong et al. (140) reported that the LC50 value at 96 h for *Tigriopus japonicus* nauplii was 0.85 mg/L for the ZnO NPs and 1.14 mg/L for ZnSO_4_ x H_2_O. Park et al. (94) indicated that the LC50 value at 96 h for adult *T. japonicus* organisms was 2.44 mg/L.

There are no acute toxicity data on *Corophium insidiosum* in the literature; the only existing data on the species refer to a chronic toxicity assessment for *Corophium volutator*, which shows that exposure to sublethal concentrations of ZnO NPs [0.2–1.0 mg/L] delays growth and influences the reproductive outcome of the exposed populations.

Previous work related to the acute toxicity of ZnO NPs to other marine amphypod crustaceans. Wong et al. (140) reported LC50 values of 1.19 mg/L for *Elasmopus rapax* exposed to ZnO NPs and 0.80 mg/L for those exposed to ZnSO_4_H_2_O. Poynton (115) reported that the LC50 values at 96 h for *Hyalella azteca* were lower than those obtained by us (0.08 mg/L for the ZnO NPs and 0.154 mg/L for ZnSO_4_), placing this species as one of the most sensitive to ZnO NPs.

Similar results to those obtained in the present study were found in the acute toxicity tests of ZnO NPs for the microalgae *Phaeodactylum tricornutum* [EC50 = 1.09 mg/L] and *Dunaliella tertiolecta* [EC50 = 1.94 mg/L] and the freshwater *cladocerus* crustacean *Daphnia magna* [LC50 = 3.2 mg/L] (135, 141, 142). Several studies on the ecotoxicity of ZnO NPs suggest that different mechanisms/modalities of action may be involved. However, most of these studies reported that the dissolution of the ionic zinc NPs contributes mainly to the toxicity of the observed ZnO NPs, and this deduction is generally based on the comparable toxicity results obtained with ZnO NPs and zinc salts (85). However, the extent to which dissolved Zn^2+^ contributes to the toxicity of ZnO NPs and the mechanisms involved are still not clear (85). The effects highlighted could be of mechanical origin: the adhesion of NP aggregates to the exoskeleton of crustaceans can in fact have physical effects, such as the obstruction of the respiratory tract and/or the loss of mobility (143, 144). Damage could also be linked to the ingestion of NPs; in organisms that ingest particles, the accumulation of these particles can be observed in the digestive tract, as we have seen for *Daphnia magna* and *Artemia salina* (116, 143, 144). The production of ROS by visible light and the generation of oxidative stress are the main mechanisms proposed to explain the negative effects of NP toxicity (145). ROS and oxidative stress damage lipids, carbohydrates, proteins and DNA, resulting in cell death (146).

Some limits of this study concern the possible aggregation/agglomeration of ZnO particles across the concentrations applied in the medium, the effective size of the agglomerate may control biological reactivity in the system, and the size distribution of the particles will be a function of concentration, and time of exposure in the medium.

Other aspects unknown or unsolved here referee to oxidative stress generation—ROS mediated mechanism—effects on enzymatic and non-enzymatic controls on ROS, internalization and distribution of NPs such as compartmentalization, bioaccumulation and histopathological alterations (91, 113–115, 131).

## Conclusion

The aim of the present work was to evaluate the acute toxicity of ZnO NPs on marine invertebrates, since at nowadays few studies on marine organisms are available. For this reason, became important to identify the Effect/Lethal Concentrations values to calculate mortality thresholds and assess potential environmental hazard. In order to allows to establish preliminary the concentrations range that can be considered toxics. These information are the fundamental importance. However, in this preliminary study, that aim only to evaluate the acute toxicity of ZnO NPs, the authors did not consider studying any mechanistic aspects of the toxic effects. It is well-known that the oxidative stress is a common pathway of toxicity induced by pollutants with the induction ROS generation and subsequent oxidative stress, which leads to damaged DNA, lipids, and proteins and potentially to cell death as reported for several organisms. Accordingly, for a full comprehension on ecotoxicity of ZnO NPs toward these marine invertebrate (still unexplored) these focus represent aims of a future study and all the limits highlighted in these preliminary results are being resolved in the study still in progress with special reference to physicochemical analyses.

More investigation, with standard experimental conditions is needed to better understand ZnO NPs toxicity to the cellular and organisms level as these NPs may enter the food chain. Environmental and human exposure due to nanomaterial residues in air, soil and crops is expected to increase with exposure routes, including possible bioaccumulation in the environment and food chain.

The possible risks, together with other unforeseen events for human health and environmental degradation, cannot be set aside or underestimated. As a consequence, effectively evaluating the specific or generalized risks associated with the various types of NPs using a precautionary approach (“no data, no market”) is necessary.

In particular, much effort should be made to develop innovative, green, and sustainable solutions (nano), which have eco-sustainability features such as limited environmental diffusion and no toxic effects on man and nature. To this end, the following measures are necessary: to support research and innovation for the identification of nanoeco-sustainable solutions; and to define a standardized methodology to evaluate the effectiveness, eco-safety and economic sustainability of NMs.

## Data Availability Statement

The datasets generated for this study are available on request to the corresponding author.

## Author Contributions

GF, EP, LV, and DC: conceptualization. EP and MT: investigation. EP, DC, MT, and AC: data curation. GF and EP: formal analysis and methodology. LV: supervision. GF, DC, and LV: writing—original draft. GF, DC, LV, AC, and LD: writing—review and editing.

## Conflict of Interest

The authors declare that the research was conducted in the absence of any commercial or financial relationships that could be construed as a potential conflict of interest.
